# Transient expansion of activated CD8^+^ T cells characterizes tuberculosis-associated immune reconstitution inflammatory syndrome in patients with HIV: a case control study

**DOI:** 10.1186/1476-9255-10-21

**Published:** 2013-05-20

**Authors:** Enrique Espinosa, Dámaris P Romero-Rodríguez, María-Teresa Cantoral-Díaz, Gustavo Reyes-Terán

**Affiliations:** 1 Center for Infectious Diseases Research (CIENI), Instituto Nacional de Enfermedades Respiratorias “Ismael Cosío Villegas”, Calzada de Tlalpan 4502, 14080 México, D.F.Mexico; 2 Present address: Laboratorio de Inmunobiología y Genética, Instituto Nacional de Enfermedades Respiratorias “Ismael Cosío Villegas”, Calzada de Tlalpan 4502, 14080 México, D.F.Mexico

**Keywords:** Activation, CD8 T cells, Highly active anti-retroviral therapy (HAART), Human immunodeficiency virus (AIDS), HIV-1, HIV-2, Inflammation

## Abstract

**Background:**

CD4^+^ T cell activation indicators have been reported to be a common phenomenon underlying diverse manifestations of immune reconstitution inflammatory syndrome (IRIS). However, we have found that a high frequency of circulating CD8^+^ T cells is a specific risk factor for mycobacterial IRIS. Therefore, we investigated whether CD8^+^ T cells from patients who develop TB IRIS were specifically activated.

**Methods:**

We obtained PBMCs from HIV+ patients prior to and 4, 8, 12, 24, 52 and 104 weeks after initiating antiretroviral therapy. CD38 and HLADR expression on naive, central memory and effector memory CD8^+^ and CD4^+^ T cells were determined by flow cytometry. Absolute counts and frequencies of CD8^+^ T cell subsets were compared between patients who developed TB IRIS, who developed other IRIS forms and who remained IRIS-free.

**Results:**

TB IRIS patients showed significantly higher counts of naive CD8^+^ T cells than the other groups at most time points, with a contraction of the effector memory subpopulation occurring later in the follow-up period. Activated (CD38^+^ HLADR^+^) CD8^+^ T cells from all groups decreased with treatment but transiently peaked in TB IRIS patients. This increase was due to an increase in activated naive CD8^+^ T cell counts during IRIS. Additionally, the CD8^+^ T cell subpopulations of TB IRIS patients expressed HLADR without CD38 more frequently and expressed CD38 without HLADR less frequently than cells from other groups.

**Conclusions:**

CD8^+^ T cell activation is specifically relevant to TB IRIS. Different IRIS forms may involve different alterations in T cell subsets, suggesting different underlying inflammatory processes.

## Background

Immune reconstitution inflammatory syndrome (IRIS) occurs in a substantial proportion of patients with HIV who initiate highly active antiretroviral therapy (HAART) [1,2] and consists of the worsening or appearance of inflammatory or AIDS-defining symptoms during effective (virus-controlling) HAART [1,3]. The potential severity of IRIS has spawned research on its prediction [4-6], prevention [7,8], and treatment [9-11]. Equally important is the determination of the immune mechanisms underlying IRIS. CD4 T cell activation, as evidenced by different cell surface markers and correlates, has been found to underlie different IRIS manifestations [12], suggesting that CD4^+^ T cell activation is a pathogenic mechanism common to diverse IRIS forms. The expansion of CD4^+^ T cells that are specific to the pathogens associated with IRIS manifestations has been observed concomitant with IRIS onset, which indicates that the participation of pathogen-specific CD4^+^ T cell responses is a general mechanism of this syndrome [13]. In addition to the mechanisms that are common to all forms of IRIS, there may be processes and cell populations that are characteristic of particular IRIS manifestations; each IRIS form has unique inflammatory manifestations that are characteristic of the opportunistic infection that is paradoxically worsened or unmasked by immune reconstitution [14,15]. For example, the plasma levels of IL-18, CCL-2, and CXCL10 differed depending on whether patients developed TB IRIS without a previous episode (unmasking IRIS) or developed it as an unexpected worsening of tuberculosis (paradoxical IRIS) [5]. We have previously found that increased frequencies of blood CD8^+^ T cells specifically predict the occurrence of IRIS related to mycobacterial infections (*Mycobacterium tuberculosis* and *Mycobacterium avium* complex (MAC)) [16], suggesting that alterations in CD^+^8 T cells are characteristic of mycobacterial IRIS. To determine whether alterations in CD8^+^ T cells characterize tuberculous IRIS, we determined the absolute counts of maturation subsets of blood CD8^+^ and CD4^+^ T cells and the frequency of activated cells among them in samples from patients who were regularly monitored during the first two years on HAART. We report an expansion of activated CD8 T cells, particularly of the naive subpopulation, concomitant with TB IRIS, unlike other IRIS forms.

## Methods

### Patients

This study was approved by the institutional Science and Bioethics Review Board. Patients were a subgroup of a previously reported HIV^+^ cohort [16] that initiated HAART, and achieved viral loads below detection limits at or before 24 weeks, as well as sustaining viral control up to week 104 (Additional 1: Figure S1A). Additionally, all patients showed increases in their counts of circulating CD4^+^ T cells (Additional 1: Figure S1B). The study group included 17 patients who developed IRIS during the first six months of treatment and 17 controls who remained IRIS free. IRIS cases (17 out of 23 in the source cohort) were selected based on availability of samples (only patients with uninterrupted sampling up to week 52 or 104), and priority was given to severe systemic disabling or potentially life-threatening cases (tuberculosis, MAC, Kaposi’s sarcoma, cytomegalovirus, herpes simplex and herpes zoster). Seventeen patients were assigned to the control group, each subject having basal CD4^+^ T cell counts in the same order of magnitude of a case, and all controls spanning the same range of values as the cases. The control group did not differ from the IRIS group in basal CD4^+^ T cell counts or viral load (Table 1). Additionally, since %CD8 T cell was a risk factor for mycobacterial IRIS in the source cohort, the chosen control group had comparable basal %CD8, so that we could detect alterations in CD8^+^ T cells that were not exclusively a consequence of greater basal %CD8^+^ T cells (Table 1, Additional file 2: Figure S2).

**Table 1:** Basal clinical data of each study group, and clinical findings at IRIS onset

	**N**	**Male/female**	**Age**	**HIV ARN copies/mL**	**CD4 T cells/μl**	**%CD8 T cells**	**Onset time (days)**	**Previous infections**	**Concomitant infections**
No IRIS	17	16/1	34.6 (30.5-39.9)	5.7 (4.9-5.9)	73.3 (33.1-109.8)	69 (65.3-75)		PCP, pulmonary TB*,* hairy *leukoplakia, oral candidosis, herpes simplex, MAC, community-acquired pneumonia,* persistent diarrhea, chickenpox, herpes zoster, aseptic meningitis	Herpes zoster, molluscum contagiosum, warts, xerosis, community-acquired pneumonia, MAC
All IRIS	17	16/1	35.5 (31–41)	5.4 (5–5.8)	35 (10.5-94.5)	74 (62–80)	55 (35.8-70.5)		
TB	6	6	33.4 (27.9-35.8)	5.2 (5.1-5.7)	61.5 (5–132)	76 (73–79)	55.5 (53–56)	Chicken pox, molluscum contagiosum, PCP, pulmonary tuberculosis, oral candidosis, MAC	Recurrent chickenpox
Other IRIS	11	10/1	36 (34.4-44.6)	5.4 (5–5.9)	35 (13.8-85.5)	74 (57–84.3)	52 (31.3-97.5)		
HS	1		36	4.3	185	77	35	Condylomata, oral candidosis	Warts, condylomata
CMV	3		30.6 (30–42)	5 (5–5.4)	35 (13.25-62.75)	63 (34.5-80.3)	56 (53–72.5)	Pulmonary and ocular CMV, PCP, oral candidosis, HIV retinopathy, skin KS,	Molluscum contagiosum, warts, skin KS, rash, xerosis
HZ	3		46.9 (40.8-49.7)	5.9 (5.6-5.9)	38 (5–80)	74 (48.3-74)	133 (111.3-178)	Molluscum contagiosum, PCP, MAC, cheilosis	*Community-acquired pneumonia*, cheilosis
KS	1		35	6	90	55	41	Chickenpox	Eczema, xerosis
MAC	3	2/1	35.2 (34.5-39.1)	5.4 (4.8-5.8)	12 (6.8-17.3)	86 (80.8-91.3)	28 (21.3-29.5)	PCP, chickenpox, KS, CMV retinitis, molluscum contagiosum	CMV retinitis, perivascular dermatitis

### IRIS definition criteria

IRIS was defined as the appearance of signs or symptoms consistent with inflammation, new opportunistic infections or the worsening of previously controlled infections during HAART. The symptoms could not be attributed to a newly acquired opportunistic infection or to drug side effects [17-22]. IRIS cases included 1 that manifested as herpes simplex retinal necrosis, 3 cytomegalovirus retinitis, 3 herpes zoster, 1 skin Kaposi’s sarcoma, 3 MAC infections (lymph node), and 6 tuberculosis (TB) (Additional file 3 Table S1). TB IRIS patients comprised four cases of unmasking and two of them manifested as paradoxical worsening (Additional file 3: Table S1). All MAC IRIS and herpes zoster IRIS cases were unmasking IRIS. In addition to reaching undetectable viremia at or before week 24 (Espinosa 2010) (Additional file 1: Figure S1A), IRIS cases and controls showed sustained increases in CD4^+^ T cell counts (Additional file 1: Figure S1B).

Eligibility for HAART initiation in the source cohort followed contemporary international guidelines [23,24]. At the initiation of HAART, patients lacked evident inflammatory processes and were either in the maintenance phase of anti-tuberculosis treatment or on effective treatments for other opportunistic infections. Prior to the initiation of HAART, active tuberculosis was ruled out in patients using the criteria of resolution by treatment; i.e., resolution of fever, cough, sputum and dyspnea, improvement of opacity, nodules, cavitations and pleural effusion, resolution of lymph node enlargement and absence of thoracic rales and dullness to percussion. Clinical findings supporting tuberculosis-IRIS (TB-IRIS) diagnose are summarized in Additional file 3: Table S1.

### Samples and staining

Blood samples were collected after clinical evaluation at each follow-up visit (immediately before HAART initiation and 4, 8, 12, 24, 39, 52 and 104 weeks after the initiation of treatment). Peripheral blood mononuclear cells (PBMCs) were obtained by density sedimentation (Lymphoprep, Oslo, Norway), cryopreserved, and thawed following the ACTG Consensus Protocol [25]. Cells were washed in medium and incubated overnight at 37°C and 5% CO_2_ prior to staining. A total of 10^6^ cells were washed with phosphate-buffered saline (PBS) containing 1% bovine serum albumin (EMD Biosciences, Darmstadt, Germany) and 0.1% sodium azide (JT Baker, Mexico). Cells were surface stained with either APC-Cy7-anti-CD4 or APC-Cy7-anti-CD8 plus APC-anti-CD45RA, PE-Cy7-anti-CCR7, PerCP-Cy5.5-anti-CD38, FITC-anti-HLADR (all from BD Biosciences, San Jose, CA), and biotin-anti-CD28 followed by Streptavidin-PE-Texas Red (BD Biosciences, San Jose, CA). Control cells were stained with anti-CD4 or anti-CD8 antibodies plus fluorochrome-conjugated isotype controls (all from BD Biosciences). Cells were re-suspended in PBS with 1% paraformaldehyde (Sigma Aldrich, Steinheim, Germany) and analyzed on a FACSAria flow cytometer (Becton Dickinson, San Jose, CA).

### Data analysis

Data were analyzed using FACSDiva (Becton Dickinson, San Jose, CA). CD4^+^ and CD8^+^ T cells were identified according to their light-scattering properties and high CD4- or CD8-associated fluorescence. Naive (CD45RA^+^ CCR7^+^), central memory (CM) (CD45RA^-^ CCR7^+^) [Lanzavecchia, Sallusto 1990, Schwendeman, Lederman], and effector memory (EM) (CD45RA^-^ CCR7^-^) cells were delineated as defined elsewhere [26-32] (Additional file 4: Figure S3). The CCR7^+^ gate was additionally verified using the FACSDiva auto-gate tool and by back-gating CD28^+^ cells into CCR7^+^ populations. Frequencies of activated subsets were determined according to their patterns of HLA-DR and CD38^+^ expression (CD38^+^ HLADR^-^, CD38^+^ HLADR^+^, and CD38^-^ HLADR^+^). (Additional file 4: Figure S3). Absolute counts of cells from different subpopulations were calculated using their proportions and the corresponding absolute CD4^+^ or CD8^+^ T lymphocyte counts. Three-group comparisons were determined with the Kruskal-Wallis test. If the Kruskal-Wallis test found overall significant differences between groups (tied p value < 0.05), post-hoc two-group comparisons were performed with the Mann–Whitney test. Tests were performed using StatView.

## Results

### Differential frequencies of activated CD8^+^ T cell subsets during HAART according to CD38 and HLADR expression

**Figure 1:**
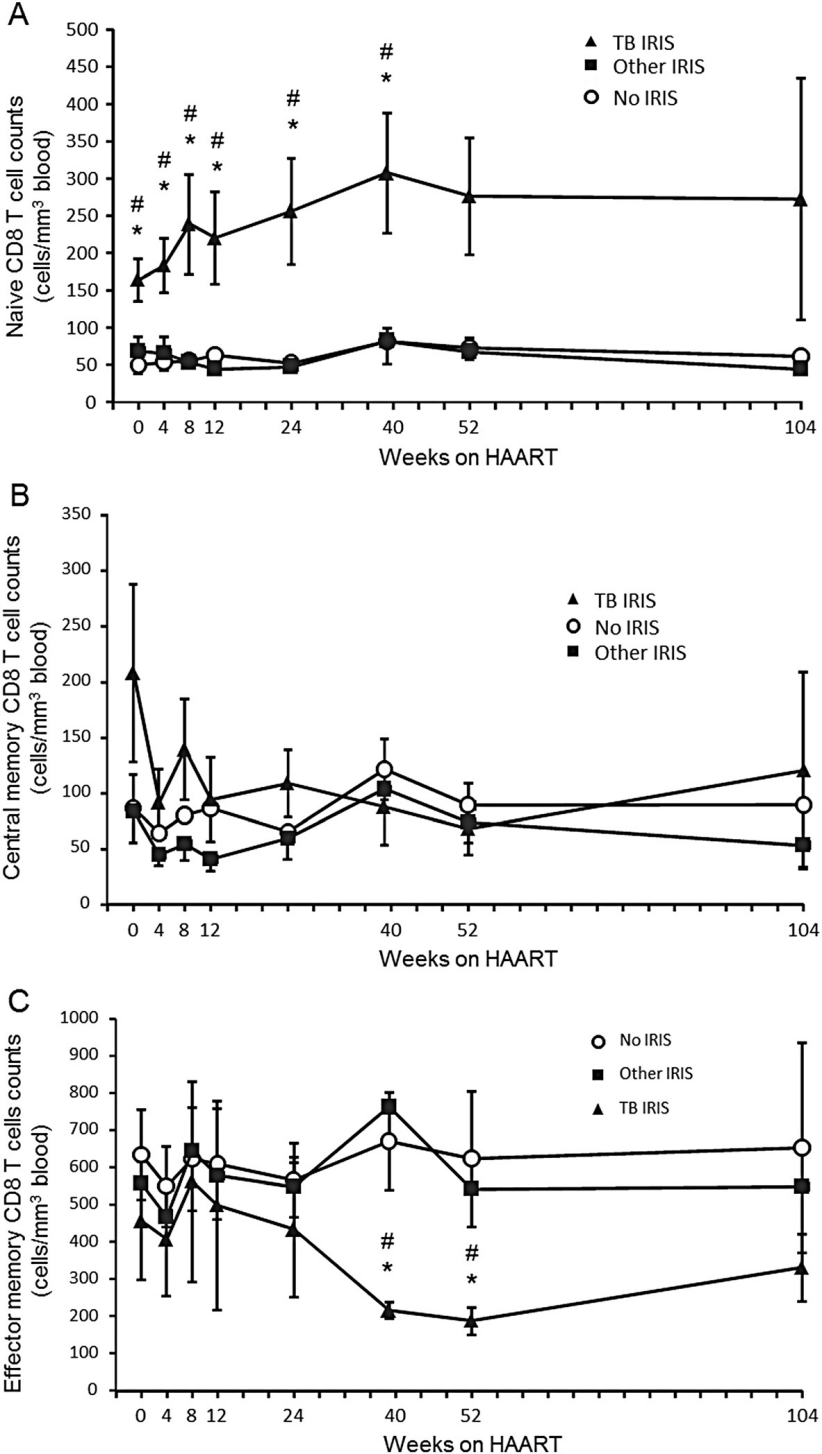
**Absolute counts of CD8 T cell subpopulations during antiretroviral treatment.** Absolute counts of naive (**A**), central memory (**B**), and effector memory (**C**) CD8 T cells before (week 0) and during antiretroviral treatment in each patient group. Values correspond to mean count ± 1 SEM. * Significant difference between the TB IRIS and No IRIS groups. # Significant difference between the TB IRIS and Other IRIS groups. Two-group differences were determined only when the Kruskal-Wallis test showed overall group effects.

The frequency of CD8^+^ T cells consistently tended to smaller decreases in the TB-IRIS group under HAART, even though all groups had comparable %CD8^+^ T cell values at week 0 (Additional file 2: Figure S2A). Groups’ %CD8^+^ T cells tended to differ at weeks 8, 12, and 24 (p = 0.0194, p = 0.062, p = 0.101, correspondingly), and showed significant differences at weeks 39 and 52 (p = 0.024, 0.014), with TB-IRIS group showing the highest percentages. These differences were parallel to differences in CD8^+^ T cell absolute counts (Figure 1B), which did not differ at HAART initiation, but significantly increased in the TB IRIS group only by week 8 (p = 0.028). CD8^+^ T cell counts differed between groups at weeks 24 and 39 (p = 0.017, p = 0.028 respectively, Kruskal Wallis), with TB IRIS group showing greater CD8^+^ T cell counts than No IRIS and Other IRIS groups at week 24 (p = 0.005, p = 0.023 respectively, Mann Whitney), and greater counts than NO IRIS group at week 39 (p = 0.0054).

**Figure 2:**
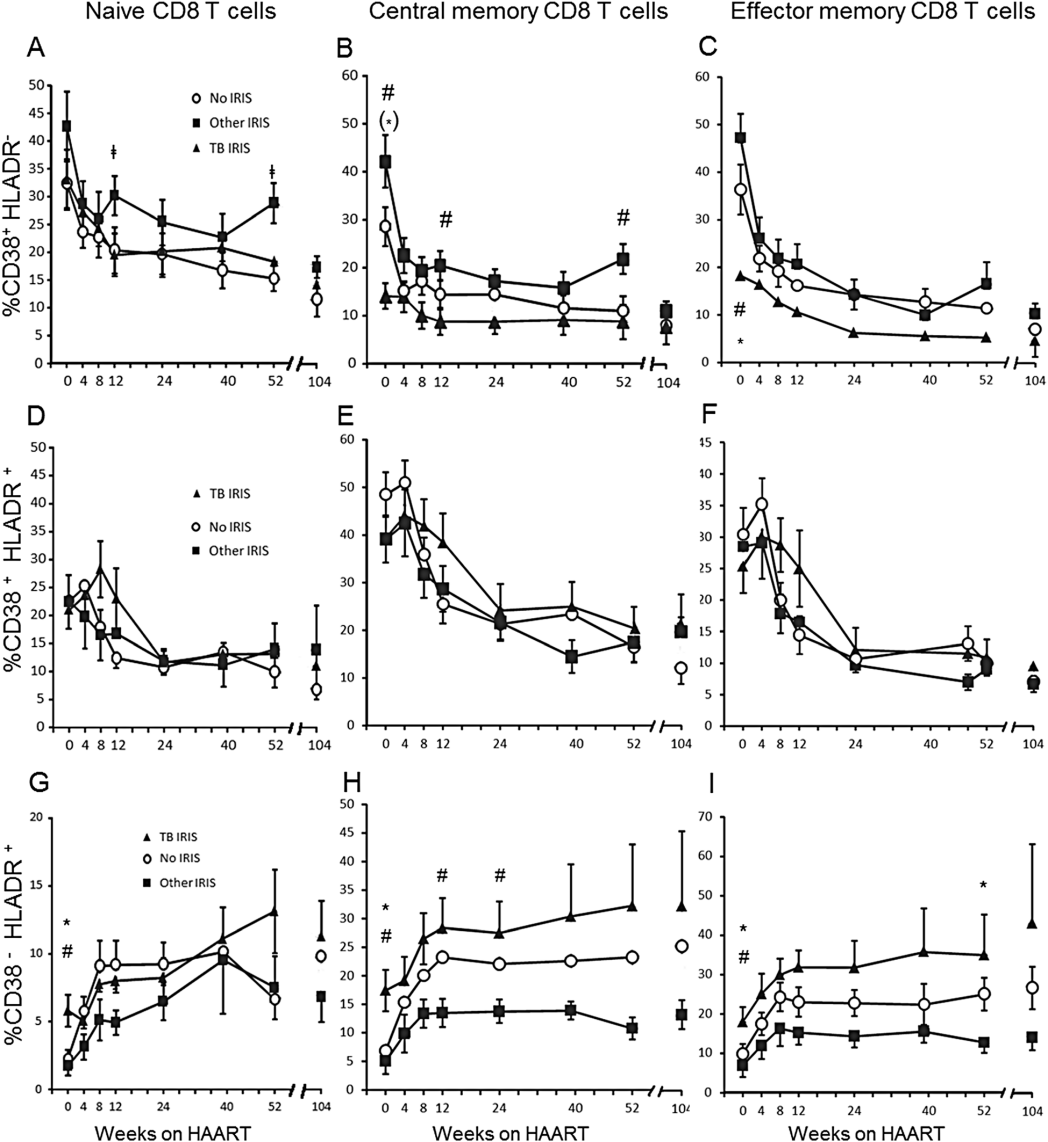
**Different patterns of CD38 and HLADR expression within CD8**^**+ **^**T cell maturation subpopulations.** Percentage of CD38^+^ HLADR^-^ cells among naive (**A**), CM (**B**), and EM (**C**) CD8^+^ T cells. Percentage of CD38^+^ HLADR^+^ cells among naive (**D**), CM (**E**), and EM (**F**) CD8 T cells throughout the study. Percentage of CD38^-^ HLADR^+^ cells among naive (**G**), CM (**H**), and EM (**I**) CD8^+^ T cells. * Significant difference between the TB IRIS and No IRIS groups. ^#^ Significant difference between the TB IRIS and Other IRIS groups. ‡ Significant difference between Other IRIS and No IRIS groups. Symbols in brackets denote tendencies (p < 0.1). Two-group differences were determined only when the Kruskal-Wallis test showed overall group effects. Values correspond to each group mean ± 1SEM.

An analysis of the frequency of activated subpopulations within CD8^+^ T cell maturation subpopulations showed peculiarities in the TB IRIS group. The TB IRIS group had lower initial frequencies of CD38^+^ HLADR^-^ cells among CM CD8^+^ T cells than the Other IRIS group (p = 0.0071, Figure 2B) and lower initial proportions of CD38^+^ HLADR^-^ among EM CD8^+^ T cells than the Other IRIS and No IRIS groups (p = 0.005 and p = 0.047, respectively; Figure 2C). In contrast, the TB IRIS group had higher initial percentages of CD38^-^ HLADR^+^ cells than the Other IRIS and No IRIS groups, whether among naive (p = 0.016 and p = 0.011, respectively; Figure 2G), CM (p = 0.01 and p = 0.012, respectively; Figure 2H) or EM cells (p = 0.04 and p = 0.014, respectively; Figure 2I). These differences were lost with treatment due to a decrease in the frequencies of CD38^+^ HLADR^-^ and CD38^+^ HLADR^+^ cells among all subpopulations and an increase in the proportion of CD38^-^ HLADR^+^ cells in all subpopulations.

TB IRIS patients were not distinguished by differential frequency of any activated CD4^+^ T cell subpopulation (Additional file 5: Figure S4). There was an overall lowering effect of HAART on the % CD38^+^ HLADR^-^ of CM and EM CD4^+^ T cells, but there were no differences between groups. The % CD38^+^ HLADR^+^ of all CD4^+^ T cell subpopulations also decreased with treatment, without differences between groups. In contrast, among naive CD4^+^ T cells, % CD38^-^ HLADR^+^ differed between groups at weeks 8 (p = 0.011), 12 (0.001), with Other IRIS having a lower % than No IRIS at these times (p = 0.004 and p < 0.001, respectively), as well as lower values than TB IRIS at week 12 (p = 0.034) (Additional file 5: Figure S4G). The % CD38^-^ HLADR^+^ of CM CD4^+^ T cells differed between groups at week 12 (p = 0.005), and 24 (p = 0.006), with Other IRIS having significantly lower % CD38^-^ HLADR^+^ of CM CD4^+^ T cells than No IRIS at these time points (p = 0.001, p = 0.002, respectively) (Additional file 5: Figure S4H). % CD38^-^ HLADR^+^ of EM CD4^+^ T cells differed between groups at week 24 only (p = 0.015), with Other IRIS values being lower than those of No IRIS (p = 0.006) (Additional file 5: Figure S4I). In contrast with CD8^+^ T cell subpopulations, no particular activation marker pattern of CD4^+^ T cell subpopulations characterized TB IRIS patients.

### Alterations in the absolute counts of CD8^+^ T cell subpopulations in patients developing TB IRIS

Absolute counts of naive CD8^+^ T cells differed significantly between groups at weeks 0, 4, 8, 12, 24, and 39 (p = 0.015, p = 0.014, p = 0.013, p = 0.022, 0.055, and p = 0.021, respectively; Kruskal-Wallis test), with significantly greater counts in the TB IRIS group than the Other IRIS and No IRIS groups at weeks 0 (p = 0.028 and p = 0.005, respectively), 4 (p = 0.014 and p = 0.005, respectively), 8 (p = 0.013 and p = 0.005, respectively), 12 (p = 0.02 and p = 0.014, respectively), 24 (p = 0.005 and p = 0.003, respectively) and 39 (p = 0.017 and p = 0.011, respectively) (Figure 1A).

There were no significant differences in absolute CM CD8^+^ T cell counts between the groups at any follow-up time point (Figure 1B). There was an apparent tendency in the TB IRIS group toward greater initial CM CD8^+^ T cell counts, which rapidly decreased upon initiation of HAART.

EM CD8^+^ T cell counts tended to decrease in the TB IRIS group during the first year of HAART (Figure 1C). Significant differences between groups were observed at week 39 (p = 0.017) and week 52 (p = 0.033), with the TB IRIS group having lower counts than the Other IRIS (p = 0.007, p = 0.027 respectively) and No IRIS groups (p = 0.017, at p = 0.014 respectively) (Figure 1C).

Subpopulation differences among CD4^+^ T cells were less evident. Naive and CM CD4^+^ T cell counts did not differ between groups (Additional file 6: Figure S5A, B). EM CD4^+^ T cell counts differed between groups only at week 39 (p = 0.036). At this follow up time, TB IRIS patients had significantly lower counts of EM CD4^+^ T cells than Other IRIS (p = 0.009), and tended to have lower counts than No IRIS patients (p = 0.09). This was the only feature of TB IRIS group regarding CD4^+^ T cell subpopulation counts (Additional file 6: Figure S5C).

### TB IRIS is characterized by a transient peak in HLADR^+^ CD38^+^ CD8^+^ T cells

**Figure 3:**
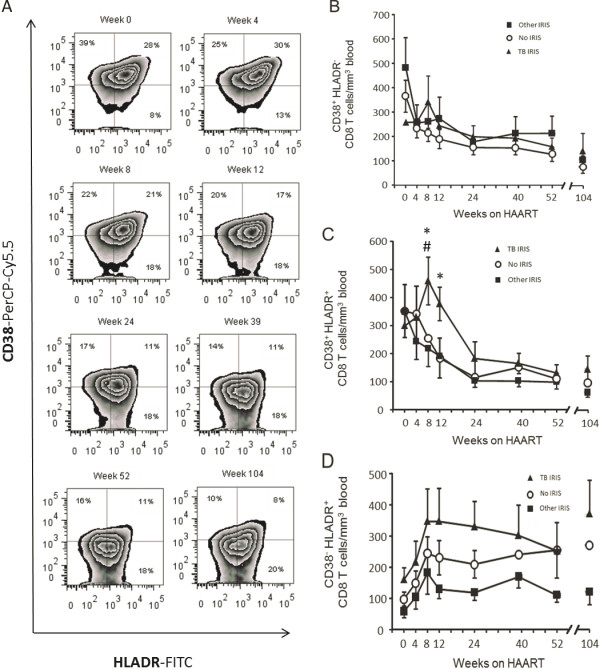
**Frequencies and absolute counts of activated CD8 T cell subsets. A**) Frequencies of activated CD8 T cell subsets according to CD38 and HLADR expression during follow up. The numbers in each quadrant correspond to the mean of all patients’ percentage of the subpopulation among all CD8 T cells. **B**) Mean ± 1 SEM absolute counts of CD38^+^ HLADR^-^ CD8 T cells. **C**) Mean ± 1 SEM absolute counts of CD38^+^ HLADR^+^ CD8 T cells. **D**) Mean ± 1 SEM absolute counts of CD38^-^ HLADR^+^ CD8^+^ T cells. * Significant difference between the TB IRIS and No IRIS groups. # Significant difference between the TB IRIS and Other IRIS groups. Two-group differences were determined only when the Kruskal-Wallis test showed overall group effects.

**Figure 4:**
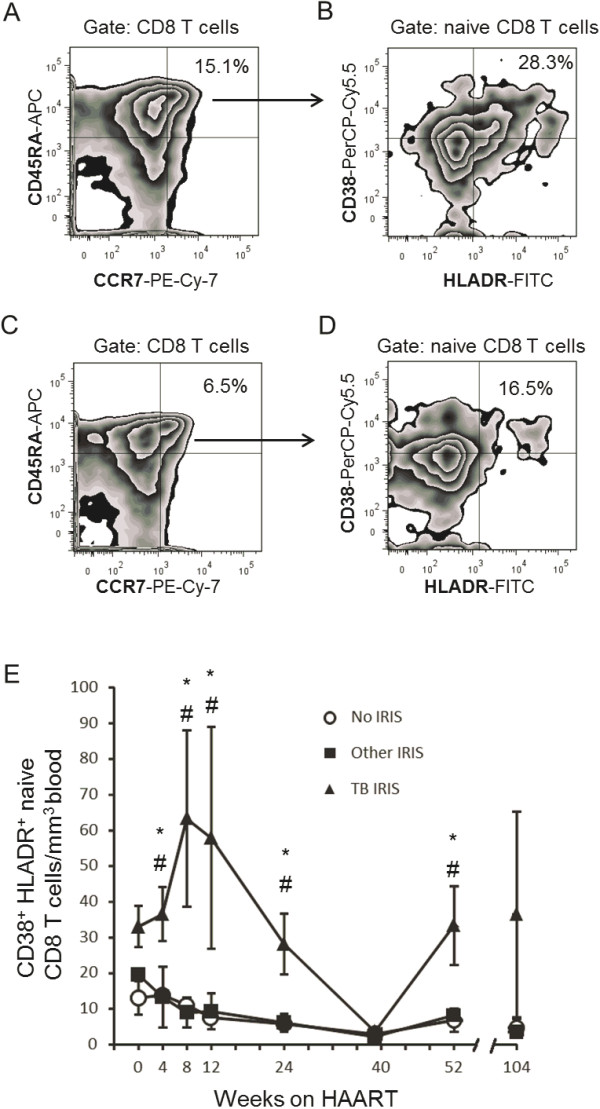
**Expansion of activated naive CD8 T cells during TB IRIS.** Zebra plots of CD8^+^ T cell maturation subpopulations according to CCR7 and CD45RA expression (see Methods) in a week 8 sample from a TB IRIS patient **(A)**, and of week 8 sample from an Other IRIS patient who developed CMV retinitis at week 8 **(C)**, showing the group’s mean % naive of CD8^+^ T cells. **(B)** Zebra plots showing activated subsets of naive CD8 T cells from the TB IRIS patient and the TB IRIS group’s mean % CD38^+^ HLADR^+^ of naive CD8 T cells at week 8. **(D)** Zebra plot showing activated subsets of naive CD8 T cells from the CMV IRIS patient and the Other IRIS group’s mean % CD38^+^ HLADR^+^ of naive CD8 T cells at week 8. **(E)** Absolute counts of CD38^+^ HLADR^+^ naive CD8^+^ T cells throughout the study. Values correspond to each group’s mean ± 1 SEM. * Significant difference between the TB IRIS and No IRIS groups # Significant difference between the TB IRIS and Other IRIS groups. Two-group differences were determined only when the Kruskal-Wallis test showed overall group effects.

The three possible expression patterns of the T cell activation markers HLADR and CD38 (CD38^+^ HLADR^-^, CD38^+^ HLADR^+^, and CD38^-^ HLADR^+^) were analyzed in CD8^+^ T cells (Figures 3 and 4). CD8^+^ T cells with each of these three patterns changed with treatment. Whereas total counts of CD8^+^ T cells expressing only CD38 began contracting as soon as HAART was initiated (Figure 3A, B), CD8^+^ T cells expressing CD38 and HLADR exhibited a delayed decrease, mainly in the TB IRIS group, resulting in significant differences between groups at weeks 8 and 12 (p = 0.025 and p = 0.028, respectively), and coinciding with TB IRIS onset (Figure 3C). At week 8, the TB IRIS group had significantly greater HLADR^+^ CD38^+^ CD8^+^ T cell counts than the Other IRIS and No IRIS groups (p = 0.039 and p = 0.006, respectively). At week 12, the TB IRIS group trended toward greater HLADR^+^ CD38^+^ CD8^+^ T cell counts than the Other IRIS group (p = 0.072), and exhibited significantly greater counts than the No IRIS group (p = 0.007) (Figure 3C). In contrast, CD8^+^ T cells expressing only HLADR were increased by HAART in both their relative frequency (Figure 3A) and absolute counts (Figure 3D).

No differences between groups in the absolute counts of CD38^+^ HLADR^-^ or CD38^+^ HLADR^+^ CD4^+^ T cells were found at any time point (not shown).

### Activated CD8^+^ T cell expansion in TB IRIS occurs in naive cells

Given that CD38^+^ HLADR^+^ CD8^+^ T cells are transiently expanded and that naive CD8^+^ T cells are significantly more numerous than other maturation subpopulations in the TB IRIS group, we calculated the absolute counts of CD38^+^ HLADR^+^ naive, CM and EM CD8^+^ T cells to determine whether an expansion of activated cells actually occurred among naive CD8^+^ T cells. Absolute counts of CD38^+^ HLADR^+^ naive CD8^+^ T cells increased after the initiation of HAART, reaching its peak at week 8; which coincided with the onset of TB IRIS (Figure 4A, B, E). This peak was not observed in patients developing other IRIS forms presenting around the same onset time (Figure 4C, D). Absolute counts of activated naive CD8^+^ T cells were significantly greater than those of the Other IRIS and No IRIS groups at weeks 4 (p = 0.02 and p = 0.014, respectively), 8 (p = 0.003 and p = 0.001, respectively), 12 (p = 0.028 and p = 0.003, respectively), 24 (p = 0.007 and p = 0.003, respectively), and 52 (p = 0.027 and p = 0.014, respectively) (Figure 4D). These differences between the groups were not observed among CM or EM CD8^+^ T cells (not shown). In the TB IRIS group, this result is exemplified by the representative week-8 plots of a patient with TB IRIS, which shows the maturation subpopulations of CD8^+^ T cells (Figure 4A) and activated cells among naive cells (Figure 4B), in contrast with the same plot from a representative Other IRIS subject (Figure 4C, D, a patient developing CMV retinitis by week 8).

## Discussion

Immune reconstitution inflammatory syndrome (IRIS) comprises a broad range of clinical manifestations, which suggests that different inflammatory processes may underlie each manifestation. At the same time, mechanisms that are common to diverse IRIS manifestations have been found, particularly CD4^+^ T cell activation [12] and a selective increase in the numbers of CD4^+^ T cells responding to the pathogen related to the IRIS manifestation [13]. In the same regard, whereas specific responses are necessary for IRIS, unspecific mechanisms such as innate mediators [5,33] may also be recruited depending on the manifestation. Consistent with the concept of characteristic mediators of particular IRIS forms, increased percentages of blood CD8^+^ T cells specifically predict mycobacterial IRIS [16]. In the present study, we report that in patients that develop tuberculous IRIS, CD8^+^ T cells have a markedly increased naive subpopulation, in which an expansion of activated cells coincides with the manifestation of TB IRIS.

An expansion of naive CD8^+^ T cells and a later decrease in EM CD8^+^ T cells were unique to TB IRIS patients during part of the follow-up period. A possible explanation to this observation could be migration of EM CD8^+^ T cells to inflamed tissues. Although cellular responses to *M. tuberculosis* antigens are commonly detected through class II MHC presentation to CD4+ T cells [34], CD8^+^ T cells also take part in the specific response to *M. tuberculosis* infection [35-39], even in the absence of CD4^+^ T cell activity [40]. In this regard, CD8^+^ T cell recruitment to the lung has been demonstrated in animal tuberculosis models [41], and by the presence of CD8^+^ T cells in pleural effusions from patients with active tuberculosis [42]. Verification of this possibility will require the detection of *Mycobacterium tuberculosis*- specific cells at the inflamed tissues of persons with TB IRIS. In any case, the expansion of naive CD8^+^ T cells in TB IRIS patients could reflect a homeostatic response to EM CD8^+^ T cell recruitment and turnover [43] and not necessarily specific responses against *M. tuberculosis*.

HAART normally decreases the frequency of CD38^+^ HLADR^+^ (activated) T cells [44,45]. However, we found an expansion of CD38^+^ HLADR^+^ CD8^+^ T cells, particularly naive CD8^+^ T cells, peaking during the presentation of TB IRIS episodes. The frequency of activated CD38^+^ HLADR^+^ CD8^+^ T cells is a strong independent predictor of HIV disease progression [46,47] and is related to inflammation in chronic HIV infection [48]. Although no mechanistic link is demonstrated here, our findings pose the question of whether activation is a driver or a consequence of IRIS and whether the control of activation might prevent IRIS [8].

Of importance, even though TB IRIS patients started HAART with a higher absolute count of total naive CD8^+^ T cells (Figure 1A), only the counts of their HLADR^+^ CD38^+^ fraction expanded after HAART (Figure 4E). The absolute counts of this activated fraction of naive CD8^+^ T cells did not differ before treatment (week 0). Therefore, the observed increase is not reflecting basal differences, but rather, a real expansion of HLADR^+^ CD38^+^ naive CD8^+^ T cells. This is also true for total HLADR^+^ CD38^+^ CD8^+^ T cells, which did not differ between groups at week 0, but transiently expanded (Figure 3B).

CD8^+^ T cell activation is not necessarily due to pathogen-specific mechanisms, as suggested by the finding that IRIS presents before the full recovery of pathogen-specific responses in both TB IRIS [49] and KS IRIS [50,51]. Moreover, because increased specific responses to *M. tuberculosis* antigens during HAART [34] are not exclusive to patients who develop IRIS, the participation of additional, less-specific mechanisms has been suggested [52,53]. Consistent with this view, soluble mediators related to both innate and adaptive immune responses have been implicated in TB IRIS [5,33].

Differences in CD4^+^ T cell subpopulation counts between patient groups were less evident likely because fewer samples were available for this determination. Additionally, we cannot rule out that recovery of CD4^+^ T cell functions was still not optimal at the time of TB IRIS events. Accordingly, patients co-infected with M. tuberculosis and HIV, in which a CD4^+^ T cell-mediated response to PPD is lowered, demonstrate elevated pleural IFN-γ levels originating from CD8^+^ T cells [54]. In a previous study of sixteen IRIS cases, among which only 2 (12.5%) manifested as tuberculosis, CD4^+^ T cell activation was demonstrated to be a feature common to the diverse manifestations of IRIS [12]. Our study included 6 TB IRIS cases among 19 IRIS cases (31.6%) and showed that CD8^+^ T cell activation was differentially increased in TB IRIS. The greater representation of TB IRIS in our study could explain our finding of CD8^+^ T cell activation patterns that are unique to this IRIS presentation.

Finally, the separate analysis of three patterns of CD38 and HLADR expression revealed phenotypes unique to TB IRIS. Patients that developed TB IRIS initiated HAART with higher frequencies of CD8^+^ T cells expressing only HLADR and reduced frequencies of cells expressing only CD38. Therefore, these subpopulations may constitute differential TB IRIS predictors and may have different functionalities.

## Conclusions

Our results suggest that in addition to common features, different cellular processes may underlie different forms of IRIS. Tuberculous IRIS is characterized by an expansion of activated CD8^+^ T cells, particularly naive CD8^+^ T cells. This finding may imply the involvement of this cellular subset in TB IRIS pathogenesis and calls for the differential treatment of IRIS manifestations.

### Consent

Written informed consent forms were approved by the Institutional Review Board. Written informed consent was obtained from the patients for research, and for publication of any report derived from it, as well as any accompanying images.

## Abbreviations

IRIS: Immune reconstitution inflammatory syndrome; CM: Central memory; CMV: Cytomegalovirus; EM: Effector memory; HAART: Highly active antiretroviral therapy; HS: Herpes simplex; HZ: Herpes zoster; IRIS: Immune reconstitution inflammatory syndrome; IQR: Interquartile range; KS: Kaposi’s sarcoma; MTB: Mycobacterium tuberculosis; MAC: *Mycobacterium avium* complex; PCP: *Pneumocystis jirovecii* pneumonia; PPD: Purified protein derivative of M. tuberculosis; TB: Tuberculosis.

## Competing interests

None of the authors has any competing interest, either financial or non-financial.

## Authors’ contributions

EE. Designed the study, designed the cytometry assays, analyzed the cytometry data, performed the statistical analysis and wrote the manuscript. DPR-R. Managed the source cohort study, obtained and cryopreserved samples, performed the cytometry assays, captured all clinical and experimental data. M-TC-D. Recorded and reviewed the clinical data, and diagnosed IRIS cases. GR-T. Designed the study, followed up the clinical data, supervised patient treatment, designed clinical data acquisition forms, defined the IRIS cases, and wrote the manuscript. All authors read and approved the final manuscript.

## Supplementary Material

Additional file 1: Figure S1 Virological and immunological responses to antiretroviral therapy. Logarithm (base 10) of the number of HIV RNA copies per mL blood at each follow up time. Values correspond to mean ± 1SEM of each group. B) Circulating CD4+ T cell counts throughout the study (number of CD4+ T cells/μL blood). Values correspond to mean ± 1SEM of each group. * Significant difference between the TB IRIS and No IRIS groups. #Significant difference between the TB IRIS and Other IRIS groups. Two-group comparisons were performed only when the three-group test showed differences between groups. Click here for file

Additional file 2: Figure S2 Higher frequencies and absolute counts of CD8^+^ T cells in TB IRIS patients. A. % CD8^+^ T cells in blood. Values correspond to each group’s mean ± 1SEM blood %CD8 T cells. * Significant differences between groups (Kruskal Wallis), with TB-IRIS group showing the greatest values. B.- Absolute counts of total circulating CD8^+^ T cells. Displayed p value was obtained with Wilcoxon’s signed rank test (weeks 0 and 8). ** Significant difference between groups with TB IRIS higher than Other IRIS and No IRIS group (p < 0.05). * Significant difference between groups with TB IRIS higher than No IRIS group. Click here for file

Additional file 3: Table S1 Diagnostic criteria and antecedents for tuberculosis associated IRIS cases. Click here for file

Additional file 4: Figure S3 Gating strategy. CD8^+^ or CD4^+^ T cells were gated according to their high CD8 or CD4-associated fluorescence and characteristic light scattering pattern. Gates for CD45RA, CCR7, CD38, and HLADR were delineated using isotype controls. Click here for file

Additional file 5: Figure S4 Different patterns of CD38 and HLADR expression within CD4^+^ T cell maturation subpopulations. Percentage of CD38^+^ HLADR^-^ cells among naive (A), CM (B), and EM (C) CD4^+^ T cells. Percentage of CD38^+^ HLADR^+^ cells among naive (D), CM (E), and EM (F) CD4^+^ T cells throughout the study. Percentage of CD38^-^ HLADR^+^ cells among naive (G), CM (H), and EM (I) CD4^+^ T cells. * Significant difference between the TB IRIS and No IRIS groups. # Significant difference between the TB IRIS and Other IRIS groups. ‡ Significant difference between Other IRIS and No IRIS groups. Symbols in brackets denote tendencies (p < 0.1). Two-group differences were determined only when the Kruskal-Wallis test showed overall group effects. Values correspond to each group mean ± 1SEM. Click here for file

Additional file 6: Figure S5 Absolute counts of CD4^+^ T cell subpopulations during antiretroviral treatment. Absolute counts of naive (A), central memory (B), and effector memory (C) CD4^+^ T cells before (week 0) and during antiretroviral treatment in each patient group. Values correspond to mean count ± 1 SEM. * Significant difference between the TB IRIS and No IRIS groups. # Significant difference between the TB IRIS and Other IRIS groups. ‡ Significant difference between Other IRIS and No IRIS groups. Two-group differences were determined only when the Kruskal-Wallis test showed overall group effects. Click here for file
