# Supplementary figures and images for: Transient expansion of activated CD8+ T cells characterizes tuberculosis-associated immune reconstitution inflammatory syndrome in patients with HIV: a case control study
Source: J Inflamm (Lond). 2013 May 20;10:21. doi: 10.1186/1476-9255-10-21 (PMC3679878; doi:10.1186/1476-9255-10-21)

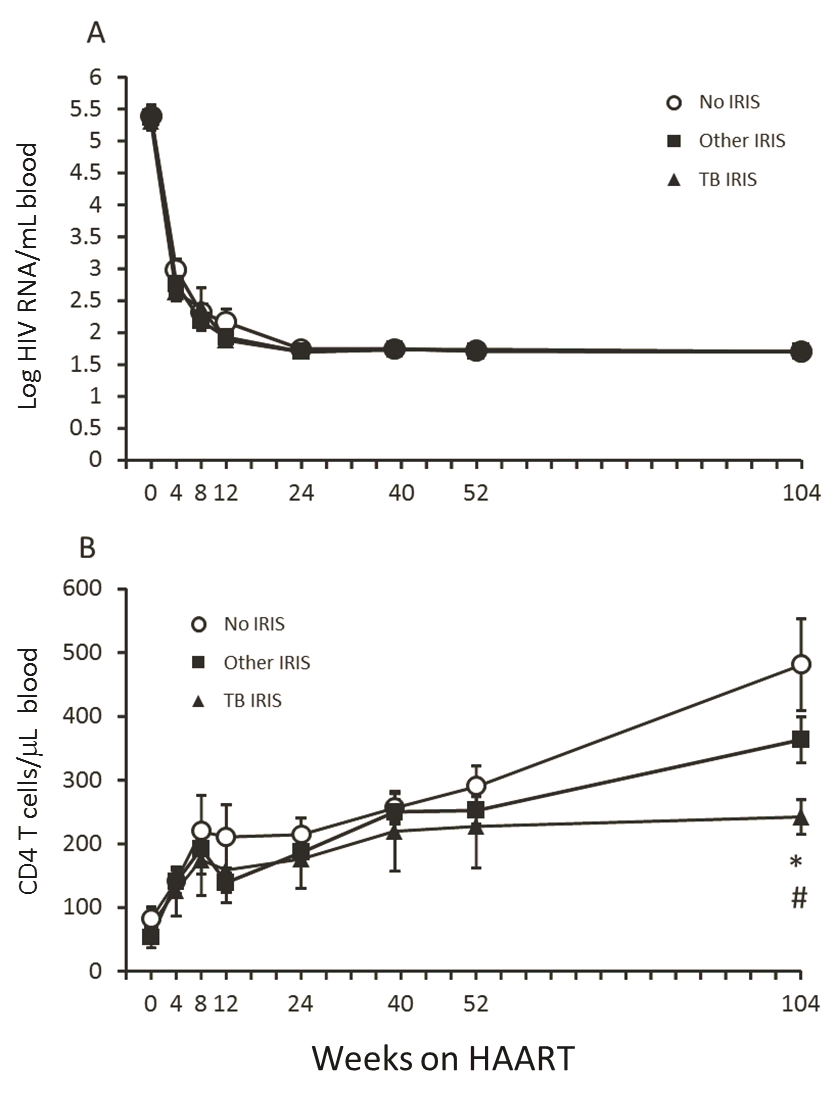

Supplement: Additional file 1: Figure S1 — Virological and immunological responses to antiretroviral therapy. Logarithm (base 10) of the number of HIV RNA copies per mL blood at each follow up time. Values correspond to mean ± 1SEM of each group. B) Circulating CD4+ T cell counts throughout the study (number of CD4+ T cells/μL blood). Values correspond to mean ± 1SEM of each group. * Significant difference between the TB IRIS and No IRIS groups. #Significant difference between the TB IRIS and Other IRIS groups. Two-group comparisons were performed only when the three-group test showed differences between groups. [file 1476-9255-10-21-S1.tiff]

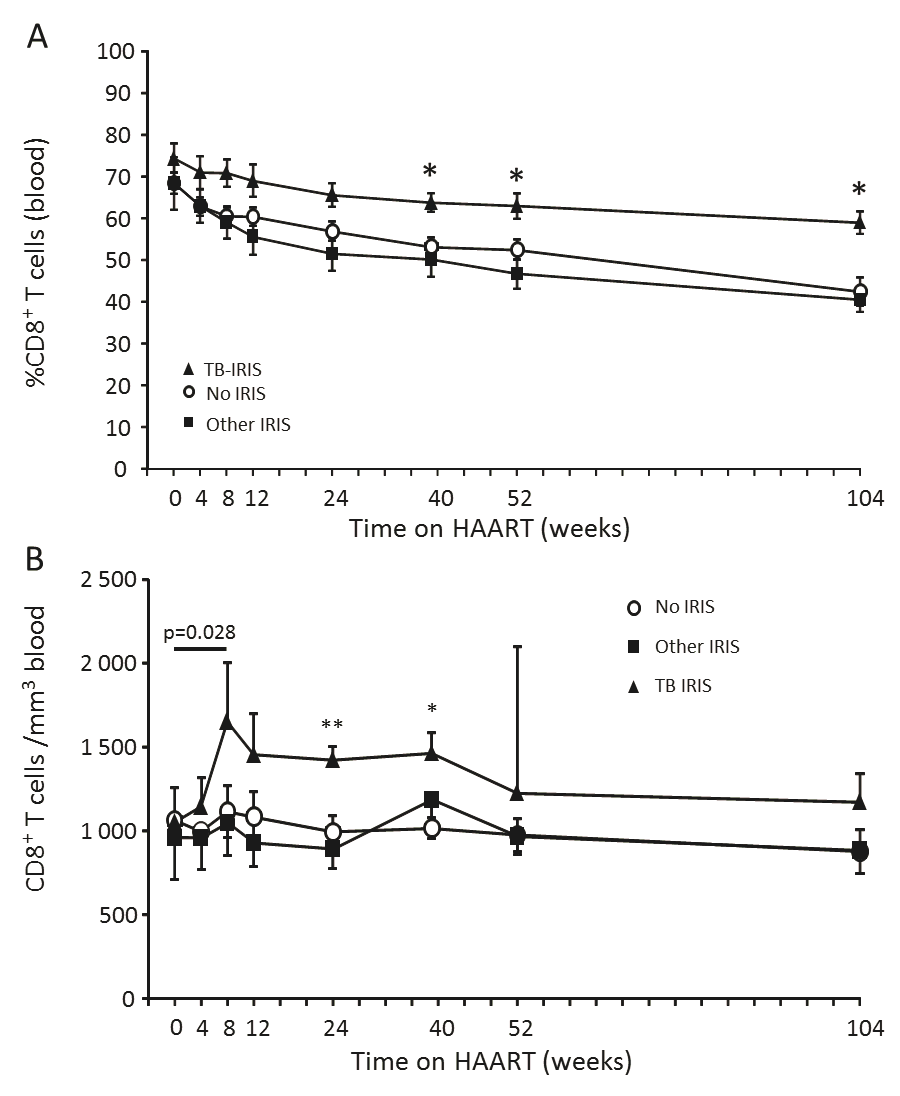

Supplement: Additional file 2: Figure S2 — Higher frequencies and absolute counts of CD8+ T cells in TB IRIS patients. A. % CD8+ T cells in blood. Values correspond to each group’s mean ± 1SEM blood %CD8 T cells. * Significant differences between groups (Kruskal Wallis), with TB-IRIS group showing the greatest values. B.- Absolute counts of total circulating CD8+ T cells. Displayed p value was obtained with Wilcoxon’s signed rank test (weeks 0 and 8). ** Significant difference between groups with TB IRIS higher than Other IRIS and No IRIS group (p < 0.05). * Significant difference between groups with TB IRIS higher than No IRIS group. [file 1476-9255-10-21-S2.tiff]

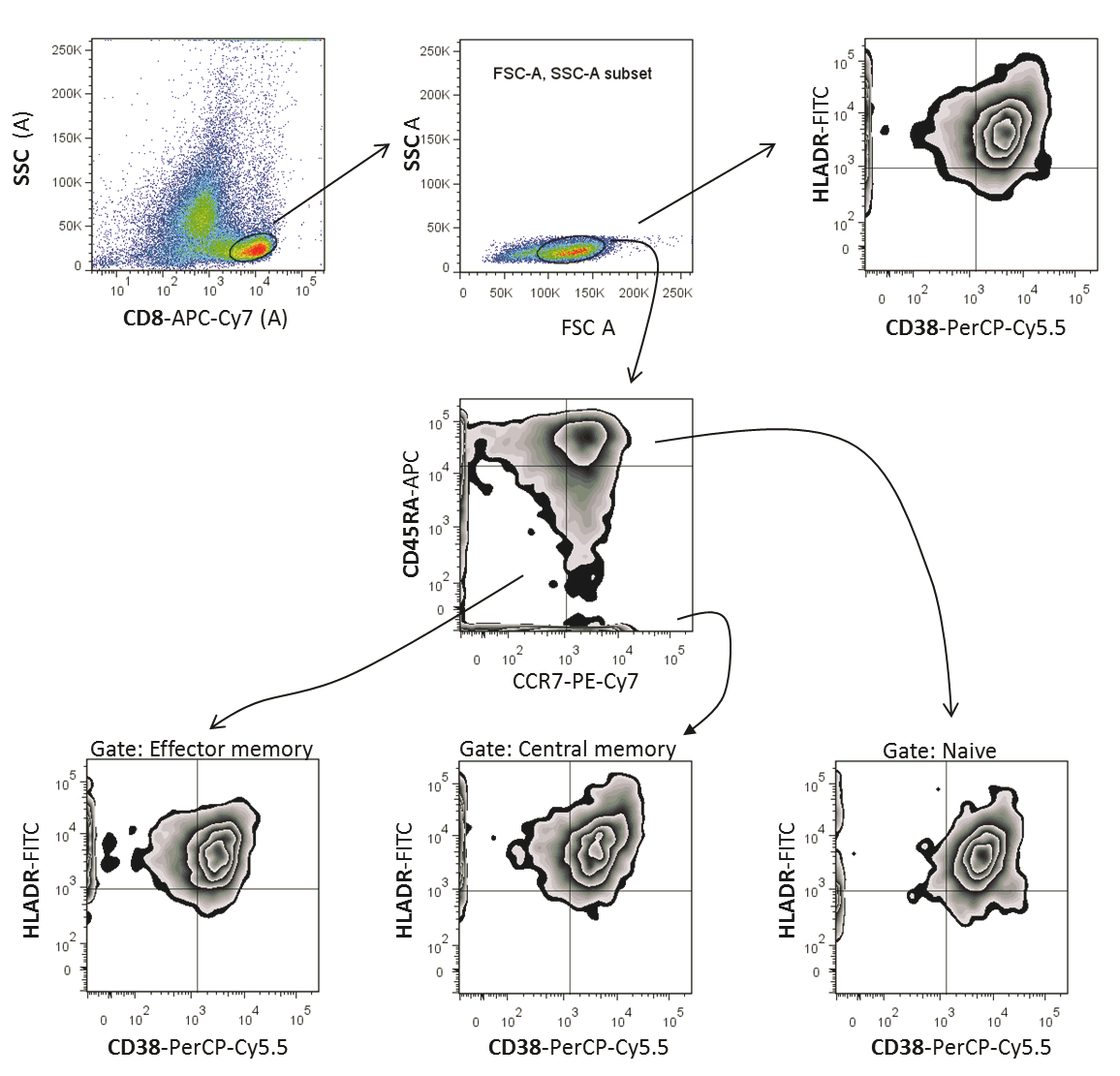

Supplement: Additional file 4: Figure S3 — Gating strategy. CD8+ or CD4+ T cells were gated according to their high CD8 or CD4-associated fluorescence and characteristic light scattering pattern. Gates for CD45RA, CCR7, CD38, and HLADR were delineated using isotype controls. [file 1476-9255-10-21-S4.tiff]

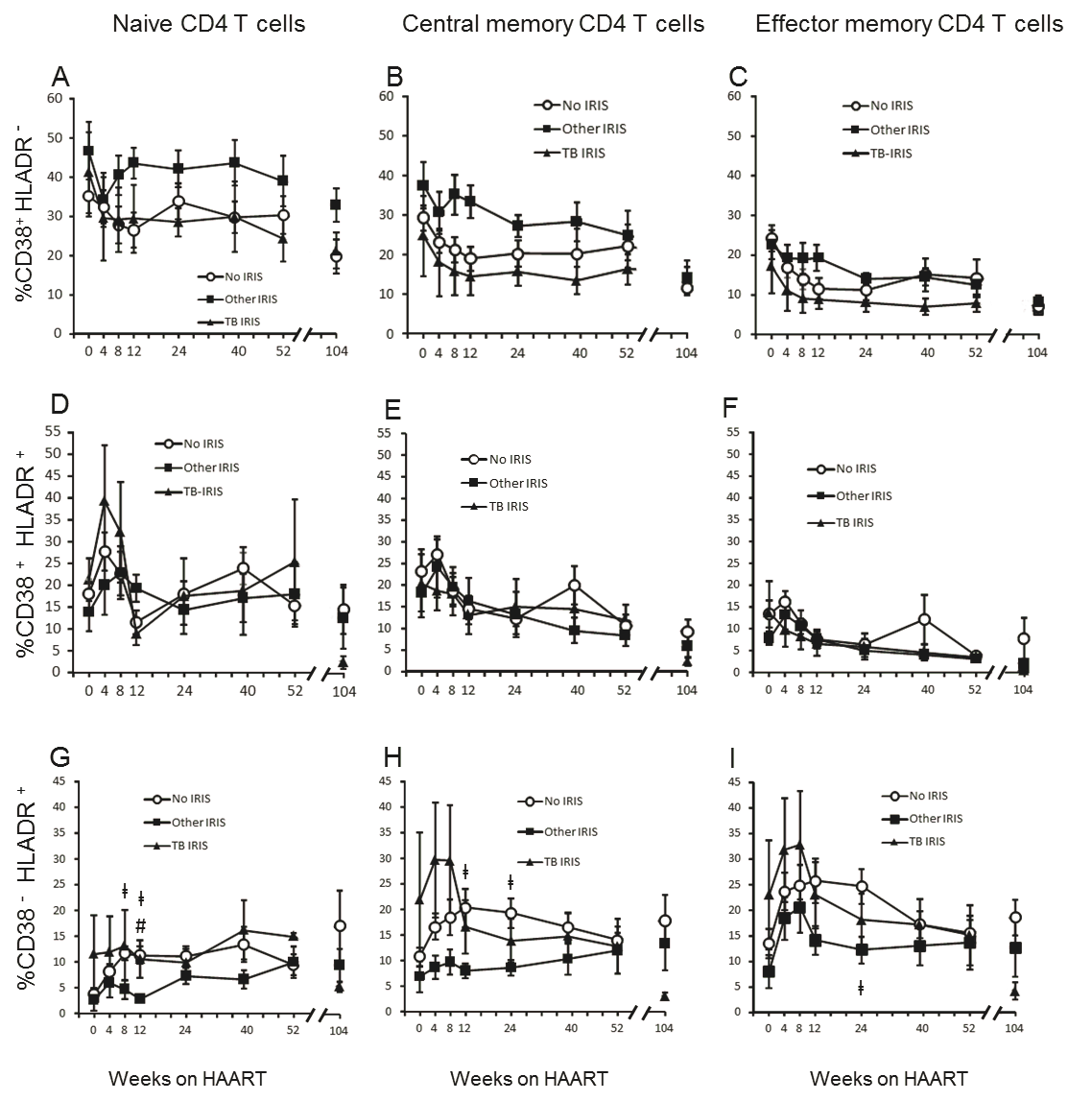

Supplement: Additional file 5: Figure S4 — Different patterns of CD38 and HLADR expression within CD4+ T cell maturation subpopulations. Percentage of CD38+ HLADR- cells among naive (A), CM (B), and EM (C) CD4+ T cells. Percentage of CD38+ HLADR+ cells among naive (D), CM (E), and EM (F) CD4+ T cells throughout the study. Percentage of CD38- HLADR+ cells among naive (G), CM (H), and EM (I) CD4+ T cells. * Significant difference between the TB IRIS and No IRIS groups. # Significant difference between the TB IRIS and Other IRIS groups. ‡ Significant difference between Other IRIS and No IRIS groups. Symbols in brackets denote tendencies (p < 0.1). Two-group differences were determined only when the Kruskal-Wallis test showed overall group effects. Values correspond to each group mean ± 1SEM. [file 1476-9255-10-21-S5.tiff]

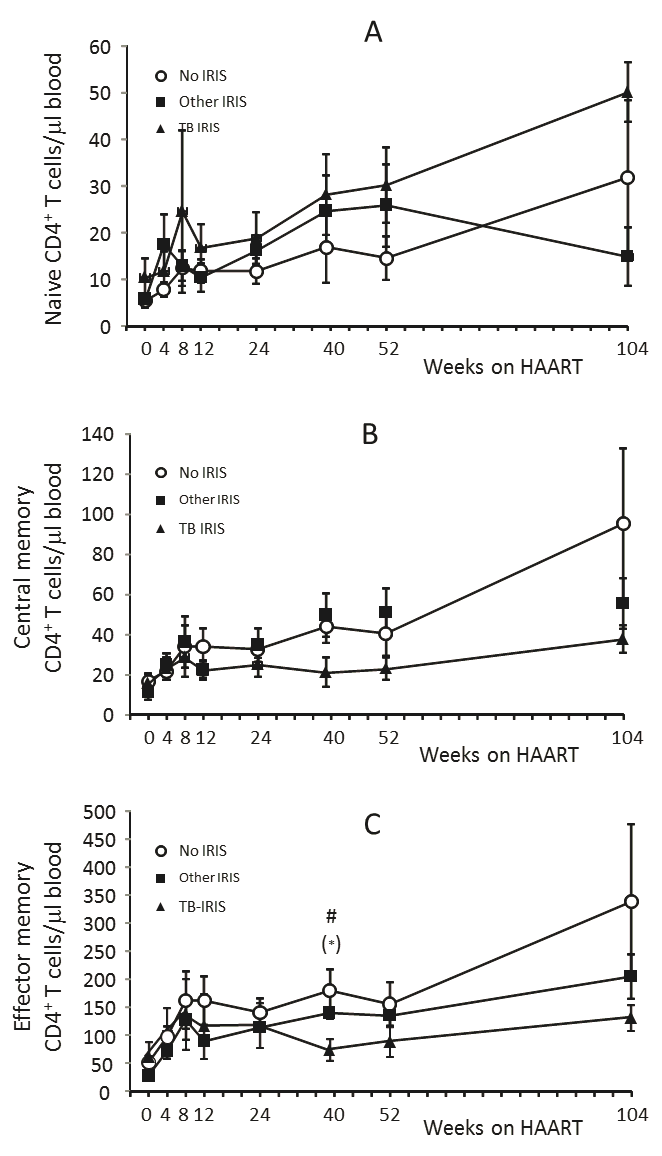

Supplement: Additional file 6: Figure S5 — Absolute counts of CD4+ T cell subpopulations during antiretroviral treatment. Absolute counts of naive (A), central memory (B), and effector memory (C) CD4+ T cells before (week 0) and during antiretroviral treatment in each patient group. Values correspond to mean count ± 1 SEM. * Significant difference between the TB IRIS and No IRIS groups. # Significant difference between the TB IRIS and Other IRIS groups. ‡ Significant difference between Other IRIS and No IRIS groups. Two-group differences were determined only when the Kruskal-Wallis test showed overall group effects. [file 1476-9255-10-21-S6.tiff]
