# Supplementary material for: Transient expansion of activated CD8+ T cells characterizes tuberculosis-associated immune reconstitution inflammatory syndrome in patients with HIV: a case control study
Source: J Inflamm (Lond). 2013 May 20;10:21. doi: 10.1186/1476-9255-10-21 (PMC3679878; doi:10.1186/1476-9255-10-21)
Supplement: Additional file 3: Table S1 — Diagnostic criteria and antecedents for tuberculosis associated IRIS cases. [file 1476-9255-10-21-S3.docx]

| Patient (institutional ID) | Antecedents | Treatments before HAART | Infections, symptoms and treatments at HAART initiation | HAART | Δ Log VL at IRIS | Δ CD4^+^ T cell/mm^3^ at IRIS | Approximate days to IRIS | Symptoms | Additional investigations or criteria | IRIS  type |
| --- | --- | --- | --- | --- | --- | --- | --- | --- | --- | --- |
| 3  (153289) | PCP pneumonia, Kleibsiella s.p. pneumonia, weight loss, oral candidosis, fever, cough, dyspnea, smoking, hairy leukoplakia, PPD-negative | TMP/SMX, Azithromycin. | TMP/SMX  No symptoms. | AZT, 3TC, EFV | -2.64 | +49 | 68 | Left cervical adenomegaly. | Granulomatous lymphadenitis with caseous necrosis. Ziehl Nielsen^+^ scant bacilli. Improvement with isoniazid, pyrazinamide, rifabutin, ethambutol. | Unmasking |
| 14  (157784) | PCP pneumonia, oral candidosis. | Azithromycin | TMP/SMX, azithromycin prophylaxis  No symptoms | AZT, 3TC, EFV | -3.42 | +85 | 53 | Cough, nocturnal fever, diaphoresis, and nausea. Heterogeneous opacity of the right pulmonary hilum (X ray). | TB^+^ BAL culture. Resistant to isoniazid and rifampicin.  Improvement with streptomycin, ethambutol, pyrazinamide and moxifloxacin. | Unmasking |
| 53  (155693) | 2005 Pulmonary tuberculosis (multi-drug resistant), control with treatment (unrecorded scheme), and relapse in 2006 (unrecorded treatment). PCP pneumonia. Oral candidosis. |  | Ethambutol, pyrazinamide, isoniazide. Moxifloxacin.  Sputum, MTB culture negative.  No symptoms | FTC, TDF, LPV/RTV | -0.55 | +51 | 56 | Painless adenomegaly. Severe headache, 39° C fever, diaphoresis, somnolence, frailty, bradilalia, dislalia, palpebral ptosis, hyporeflexia, emesis. Referral to neurology hospital. Coma, death. | Vasculitis upon post-mortem pathological examination. Abundant bacilli in brain vasculature. | Worsening |
| 62  (162141) | Chronic diarrhea, Pulmonary tuberculosis. Oral candidosis. | Rifampicin, isoniazide, pyrazinamide, ethambutol | Maintenance TB treatment (rifampicin, isoniazide, pyrazinamide)  Sputum Ziehl Neelsen stain negative.  No symptoms | TDF, FTC, EFV | -4.02 | +202 | 55 | Mesogastric colic-like pain irradiating bilaterally to iliac fosse, nausea, diaphoresis, emesis with bile. Referred to emergency service. Diiagnosis: Chronic granulomatous ileitis with perforation, compatible with TB. Chronic granulomatous colitis with ulceration and hemorrhage, chronic granulomatous peritonitis, granulomatous appendicitis, chronic granulomatous enteritis. Ileostomy (77cm) Colostomy (24 cm) | Resolved after surgery and continued rifampicin, isoniazide, pyrazinamide.  Mesenteric TB-IRIS. | Worsening |
| 69  (164802) | Weight loss.  (No additional symptoms).  Contact with relative with active tuberculosis | TMP/SMX  Isoniazid and pyridoxine prophylaxis | Isoniazid and pyridoxine,  TMP/SMX  No symptoms | TDF, FTC, EFV | -2.8 | +19 | 36 | Right cervical adenomegaly, pain. | Ziehl Nielsen stain positive. Resolution under Rifampicin, pyrazinamide, isoniazid plus ethambutol | Unmasking |
| 77  (165108(165108) | Chronic diarrhea, hairy leukoplakia.  PCP pneumonia  TB-negative (culture) lung biopsy | TMP/SMX Prednisone, ceftriaxone. | TMP/SMX  No symptoms | TDF, FTC, EFV | -2.6 | +211 | 56 | Fever, shivers, headache, lumbar pain, TB PCR^+^ cerebrospinal fluid. | Response to Rifampicin, isoniazid, pirazinamide and ethambutol | Unmasking |
